# Pain science education concepts for pelvic pain: an e-Delphi of expert clinicians

**DOI:** 10.3389/fpain.2025.1498996

**Published:** 2025-02-04

**Authors:** Amelia K. Mardon, Hayley B. Leake, Monique V. Wilson, Emma L. Karran, Romy Parker, Rinkle Malani, G. Lorimer Moseley, K. Jane Chalmers

**Affiliations:** ^1^ IIMPACT in Health, University of South Australia, Adelaide, SA, Australia; ^2^ The Pain Education Team to Advance Learning (PETAL) Collaboration; ^3^ NICM Health Research Institute, Western Sydney University, Westmead, NSW, Australia; ^4^ Persistent Pain Research Group, Hopwood Centre for Neurobiology, ifelong Health Theme, SAHMRI, Adelaide, SA, Australia; ^5^ Department of Anaesthesia and Perioperative Medicine, University of Cape Town, Cape Town, South Africa; ^6^ MGM School of Physiotherapy, A Constituent Unit of MGMIHS, Aurangabad, India

**Keywords:** pelvic pain, endometriosis, pain science education, Delphi, consensus, patient education

## Abstract

**Introduction:**

Persistent pelvic pain is a prevalent condition that negatively impacts one's physical, psychological, social, and sexual wellbeing. Pain science education (PSE) involves learning about the biology of pain and is commonly integrated into the management of persistent pain. However, PSE is yet to be thoroughly investigated for persistent pelvic pain potentially due to the lack of targeted curricula, including learning concepts. The aim of this study was to gain consensus on PSE learning concepts important for persistent pelvic pain according to expert clinicians.

**Methods:**

A three-round e-Delphi survey was conducted to generate and gain consensus on important PSE learning concepts for female persistent pelvic pain among 20 international, multidisciplinary expert clinicians (e.g., physiotherapists, gynaecologists, psychologists). Learning concepts generated by clinicians were rated by importance using a six-point Likert scale. Consensus on importance rating was considered reached for items with an IQR <1.0.

**Results:**

The expert clinicians generated 125 PSE learning concepts that were considered important for persistent pelvic pain; 92 (73.6%) learning concepts reached consensus on their importance rating. Of the 125 learning concepts, 102 were generated for persistent pelvic pain in general, and were categorised into 13 overarching PSE concepts (e.g., persistent pelvic pain involves changes to the brain and nervous system). Sixteen PSE concepts were generated for specific pelvic pain conditions (e.g., endometriosis) and seven concepts for specific life stages (e.g., adolescence).

**Discussion:**

This study provides the first list of key PSE concepts tailored for persistent pelvic pain developed by expert clinicians. These concepts provide a framework for developing and implementing PSE curricula for persistent pelvic pain in research and clinical settings.

## 1 Introduction

Persistent pelvic pain (herein referred to as “pelvic pain”) is an umbrella term for conditions associated with pain in the pelvis for more than three months and symptoms suggestive of lower urinary tract, bowel, pelvic floor, sexual, or gynaecological dysfunction (1). This study focuses on pelvic pain associated with benign gynaecological and urological conditions. Despite guidelines endorsing a biopsychosocial approach to the management of pelvic pain (2, 3), they most frequently recommend biomedical interventions (e.g., surgery, medication) (4). Limited implementation of a biopsychosocial approach to pelvic pain management may in part be due to the disconnect between this approach and someone with pelvic pain's current understanding of pain, and thus expectations regarding pain management. Pain science education (PSE) may encourage people with pelvic pain to engage with biopsychosocial-informed management strategies.

Pain science education aims to provide people with a sufficient understanding about what pain is, how it works, and why it may persist (5). Pain science education is underpinned by conceptual change theories that focus on achieving specific learning outcomes about pain biology and management (6), and reconceptualise pre-existing misconceptions about pain from a biomedical lens towards a biopsychosocial paradigm (5). Meta-analyses of randomised trials have demonstrated that PSE is effective at improving pain intensity and disability for musculoskeletal conditions, when provided alongside other active interventions (7, 8). Preliminary evidence suggests PSE may also be beneficial for females with pelvic pain. A pre-post study showed that a PSE seminar improves pain knowledge for females with pelvic pain (9). A case series (10) and a non-randomised clinical trial (11) that integrated PSE alongside physiotherapy also found improvements in pain intensity. However, the curricula used across these studies are varied and primarily based on pain science resources developed for other persistent pain conditions, which suggests they may not be including concepts specific to pelvic pain.

Curricula-building is a critical component for developing effective PSE resources. Considering the views of expert clinicians is important when developing such curricula because they are knowledgeable of the topic and have clinical experience delivering education (12). Including the views of people with pain (herein termed “consumers”) is also important when developing a PSE curriculum (12–15). A qualitative study has gathered the views of people with pelvic pain to investigate what PSE concepts were most important for them to learn (16). We hypothesize that a final curriculum would encompass those results alongside the concepts that are generated by expert clinicians. Therefore, the objective of this study was to generate and gain consensus among expert clinicians on important PSE concepts for females with pelvic pain.

## 2 Methods

This study was three-round electronic Delphi (e-Delphi) survey. The design and conduct of this study is reported in accordance with recommendations for the Conducting and reporting of Delphi Studies (CREDES) (17). The protocol was pre-registered on Open Science Framework on 15 August 2022 (https://osf.io/bzgwp/), with deviations noted within the manuscript. Ethical approval was obtained from the Human Research Ethics Committee of the University of South Australia (no. 204706).

### 2.1 Participants and recruitment

A purposive sample of expert clinicians were recruited to participate in the e-Delphi survey. An expert clinician was defined as someone with clinical experience treating females with pelvic pain, and who fit the following criteria: (1) held a relevant tertiary qualification in their clinical speciality (e.g., physiotherapy, medicine, psychology); (2) had > two years full-time equivalent experience treating females with pelvic pain associated with benign gynaecological and urological conditions; (3) had practiced clinically within the past two years; (4) had additional training in the contemporary understanding of persistent pain science; (5) was proficient in the English language.

We aimed to recruited a diverse range of clinicians to seek a variety of opinions, thereby increasing consensus validity (18). To ensure diversity, panel members were recruited from a range of international geographical locations, rural and metropolitan areas, age groups, professional groups (training and clinical experience), and healthcare settings (e.g., public, private). Based on the expected heterogeneity of the panel and a likely drop-out rate of 20%–30% between survey rounds (19), we decided *a priori* to invite 20 expert clinicians. We used a purposive, snowballing sampling approach to recruit eligible panel members. First, we identified potential panel members using the professional network of the authorship team and Internet searches. Panel members were also asked to identify further potential panel members through their networks (i.e., snowballing). Panel members were invited by the primary researcher (AKM) to participate by a personalised email, which contained detailed information about the study. Informed consent from each panel member was obtained electronically at the start of the Round One survey.

### 2.2 Survey development and procedure

Qualtrics software (2019, SAP, Provo, UT, USA) was used to develop, conduct, and distribute the e-Delphi survey rounds. Participation was anonymous – a key characteristic of the Delphi process (19). Prior to distribution, the Round One survey was piloted by two local experts to collate feedback on readability, relevance, and usability; edits were made accordingly.

The Round One survey link was open from 28 August 2022 to 18 September 2022, with email reminders sent twice during this period. The Round One survey comprised of three parts: (1) study information and participant consent; (2) panel members' demographics (e.g., sex, gender, country of residence, profession); and (3) open-ended questions asking their opinion on what content should be included in PSE for females with benign gynaecological and/or urological pelvic pain. Subsequent questions asked panel members to describe additional educational information specific to pelvic pain conditions (e.g., endometriosis) or life stages (e.g., adolescence).

The Round Two survey included the concepts identified in Round One survey responses. The survey link was open from 4 October 2022 to 18 October 2022 to participants who completed the Round One survey (due to consent purposes). Email reminders were sent twice during this period. Participants were invited to rate the importance of each concept in a randomised order using a six-point Likert rating scale (“Not at all important” - “Very important”). Concepts were presented in three parts: (1) PSE concepts important for females with pelvic pain; (2) PSE concepts important for specific pelvic pain conditions; (3) PSE concepts important for specific life stages. Open-ended questions were provided following each survey section to allow panel members to provide further comments on each concept (e.g., suggested rewording of items, condensing multiple statements into one) and identify any further concepts for consideration into the Round Three survey.

Following Rounds Two and Three, panel members were given controlled feedback of survey responses, including their individual responses and the group median and interquartile range (IQR) on each survey item. Feedback was also provided for open-ended responses, which included clear delineation of reworded or removed statements and reasoning behind the changes and labelling of any new statements generated. The purpose of this feedback was to allow panel members to reflect and potentially revise their responses when compared to the groups response, with the aim of reaching consensus by the end of Round Three (19) (see data analysis section for consensus criteria).

The Round Three survey comprised of concepts rated in the Round Two survey, including those that were re-worded and new concepts suggested. The Round Three survey link was open from 31 October 2022 to 14 November 2022 to panel members who completed the Round One survey. Email reminders were sent twice during this period. The Round Three survey was conducted in the same manner as Round Two.

Although Delphi survey rounds can be performed until consensus is reached, panel members' responses are unlikely to change following three rounds of rating statements (19). Thus, we decided *a priori* that we would conduct a maximum of three survey rounds after generation of statements (a total of four rounds for this study) unless stability of survey responses was reached in earlier rounds. Following termination of the e-Delphi process, the final list of concepts (derived from Round Three survey) was grouped into categories by the first author (AKM) before being discussed and refined with the wider research team. Panel members were then invited via email to provide anonymous feedback on the grouping of concepts and the developed categories using Google Docs software (California, USA) or email. Feedback was collected from 12 January 2023 to 26 January 2023; two email reminders were sent during this time, which was a deviation from the protocol to allow panel members more time to provide feedback.

### 2.3 Data analysis

Quantitative data were analysed using IBM SPSS v26 (20). Descriptive statistics were used to analyse demographic data and survey response rates. Survey item scores were analysed by median and IQR because they are robust and objective measures of consensus, which can be given pre-defined cut-off values (19, 21). For all survey rounds, items were retained if they were considered “important” (a median >3.0 - “slightly important”); items were excluded if they were rated “unimportant” (median value of <3.0) and had reached consensus (IQR <1.0). Consensus was considered reached between panel members for items with an IQR <1.0. Stability of responses between survey Rounds Two and Three were calculated using the nonparametric Wilcoxon matched pairs signed rank test (19, 21). Panel members' responses were considered stable when no statistically significant change (*p* ≥ .05) was detected between the rounds (21). The final list of PSE concepts (“items”) were ranked within their defined categories based on their median Likert scale rating of importance.

Qualitative data were analysed using QSR International's NVivo software (release 1.6.1). For Round One, open-ended responses were analysed using a simple inductive, content analysis method (22, 23). Two researchers (AKM and MVW) independently open-coded responses and collated their codes into representative “concepts”, taking into consideration wording used by participants (22). Responses that were the same, or very similar, between participants were combined and collapsed into a single statement, whilst ensuring to stay close to the meaning of the original suggestions. To ensure consistency and reliability between coders, inter-coder reliability was determined using Cohen's kappa. Kappa values were categorised as having no agreement (<0.20), minimal agreement (0.21–0.39), weak agreement (0.40–0.59), moderate agreement (0.60–0.79), strong agreement (0.80–0.90), and almost perfect agreement (>0.90) (24). Inter-coder reliability was piloted in a random sample of 20% of survey responses. When agreement was moderate (Cohen's kappa >0.60), coders continued open-coding the remaining responses. Open-ended responses from subsequent survey rounds, including proposed rewording of concepts and suggested new statements, were analysed by AKM and discussed among the wider research team.

## 3 Results

**Figure 1:**
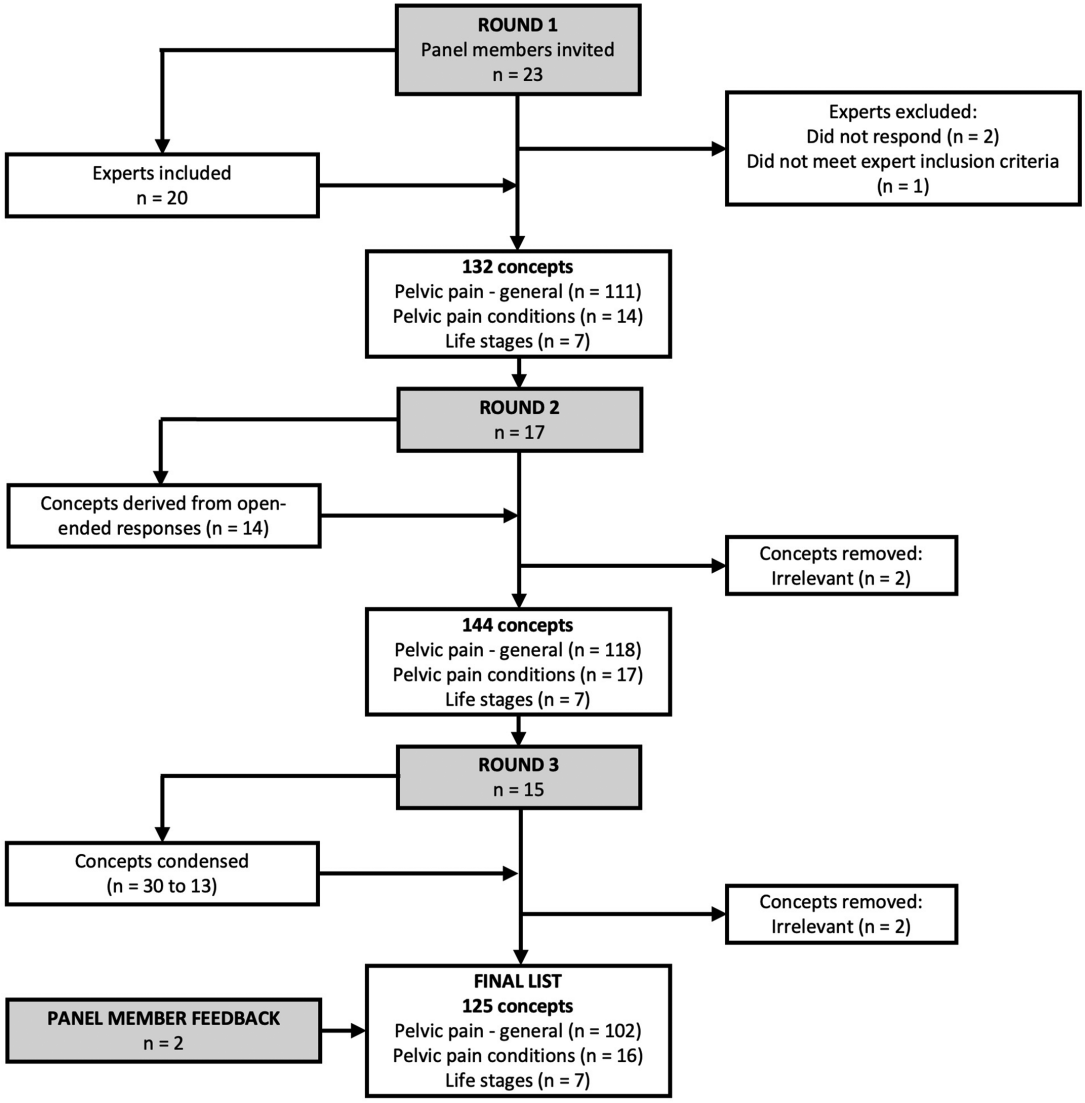
Flowchart of e-Delphi process.

Twenty-three experts were invited to participate in the e-Delphi study (Figure 1). Two experts did not respond, and one expert was excluded because they did not meet the eligibility criteria. Twenty participants took part in the Round One survey (participant demographics are presented in Table 1). Most of the expert panel were female (*n* = 18/20), born and resided in Australia at the time of the survey (*n* = 8/20), and half were physiotherapists (*n* = 10/20).

**Table 1:** Panel member demographics.

Demographics		*n* (%)
Sex	Female	18 (90)
	Male	2 (10)
Gender	Female	18 (90)
	Male	2 (10)
Country of birth	Australia	8 (40)
	South Africa	3 (15)
	USA	2 (10)
	UK	2 (10)
	Canada	1 (5)
	Spain	1 (5)
	Romania	1 (5)
	NZ	1 (5)
	India	1 (5)
Country of residence	Australia	8 (40)
	South Africa	3 (15)
	USA	2 (10)
	Canada	2 (10)
	India	2 (10)
	UK	2 (10)
	Spain	1 (5)
Region of residence	Metro	18 (90)
	Rural	2 (10)
Identify as belonging to a minoritised group	Yes	5 (25)
	No	15 (75)
Profession^a^	Physiotherapist	10 (50)
	Gynaecologist	4 (20)
	Gynaecologist and pain specialist	2 (10
	Nurse	1 (5)
	Exercise physiologist	1 (5)
	Psychologist	1 (5)
	General practitioner	1 (5)
Healthcare sector^b^	Private	15 (75)
	Public	7 (35)
Clinical experience treating PPP (years)	2–5	6 (30)
	6–10	2 (10)
	10–20	4 (20)
	20+	8 (40)

Total *n* = 20.

^a^ One expert identified as both a physiotherapist and medical doctor.

^b^ Multiple options could be selected.

### 3.1 Round one

Twenty panel members responded to the Round One survey. From the open-ended responses, 110 statements were identified relating to PSE concepts for pelvic pain. For PSE concepts specific to pelvic pain diagnoses, eight concepts were identified for endometriosis and adenomyosis, two for bladder pain syndrome (BPS), four for vulvodynia and vulvar pain, and one for sexual pain. For PSE concepts specific to life stages, four concepts were identified for adolescents, one for reproductive years, and two for post-menopausal pelvic pain. See Supplementary File 1 for content analysis inter-coder reliability.

### 3.2 Round two

Seventeen panel members responded to the Round Two survey. Panellists were provided 132 concepts to review. All concepts were rated as “important” (median >3.0) and 109 (82.6%) reached consensus on their importance rating (IQR >1). No concepts met the exclusion criteria (i.e., unimportant). Therefore, all concepts were retained for inclusion in the Round Three survey.

Panel members suggested rewording 16 concepts and added 14 concepts for the Round Three survey. The research team also excluded two concepts that were originally included in the Round Two survey because they did not meet the study aims.

### 3.3 Round three

Fifteen panel members responded to the Round Three survey. Panellists were provided 144 concepts to review, and all were rated as “important” (median >3.0); 104 (72.2%) concepts reached consensus on their importance rating. One concept did not meet stability between rounds two and three (*p*-value <0.05). Due to the high rate of stability, the e-Delphi process was terminated, and a further round (Round Four) was not required. Stability could not be calculated for the 14 concepts suggested in the Round Two survey.

Panel members condensed 30 concepts into 13. Panel members also suggested the rewording of 11 concepts; no additional concepts were suggested. Three concepts were removed from the survey by the research team because they did not meet the study aims.

### 3.4 Final concepts and panel member feedback

There were 125 final concepts, of which, 92 (73.6%) reached consensus on their importance rating (IQR ≤1.0). One hundred and twenty-four (99.2%) concepts reached stability between Rounds Two and Three. Nine statements were re-worded by the research team for clarity and to reflect consistency with wording of other concepts.

**Figure 2:**
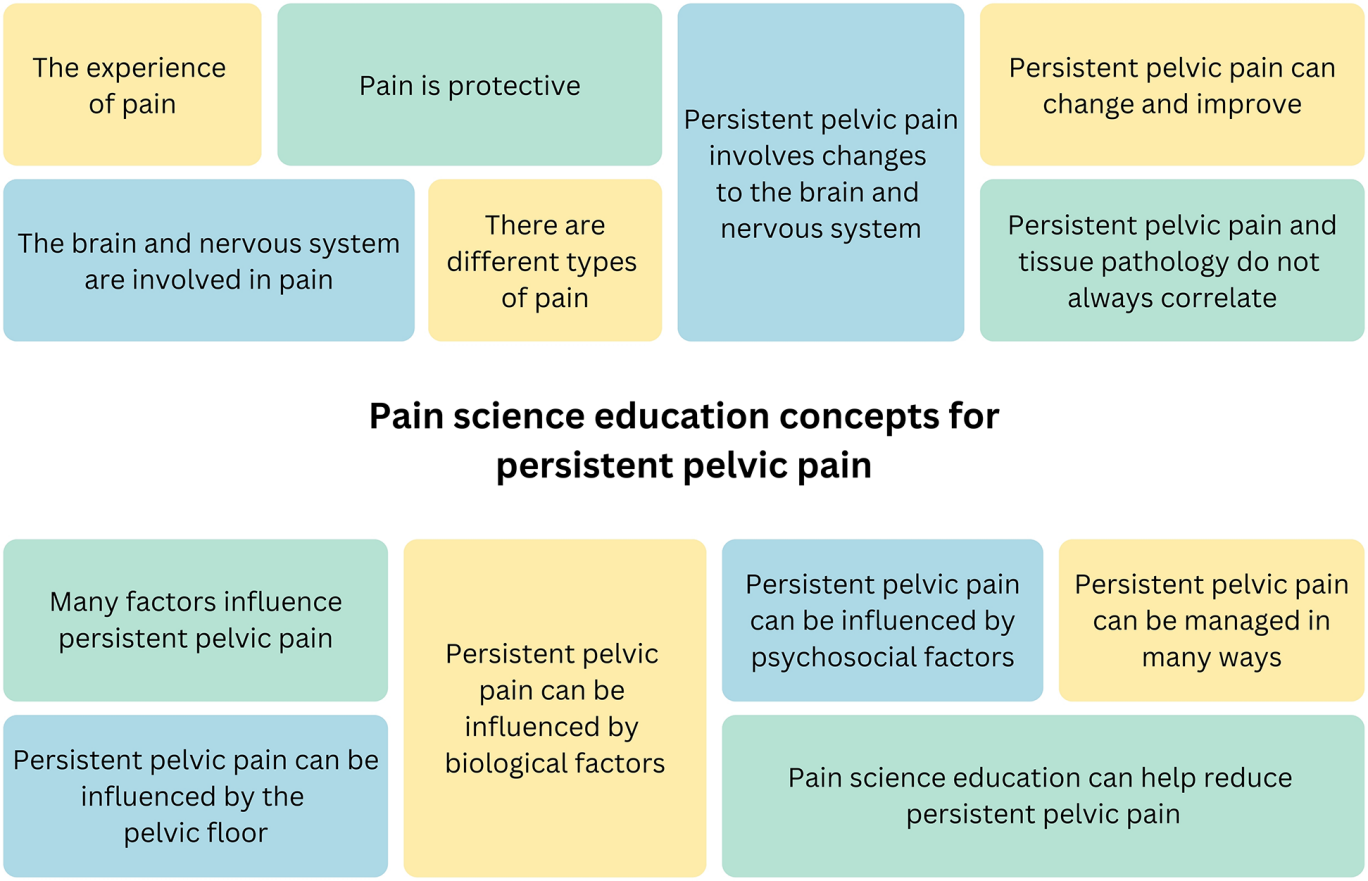
Overarching pain science education concepts generated by expert clinicians for persistent pelvic pain.

Of the final concepts, 102 were generated for female pelvic pain in general; concepts were grouped into 13 categories representing overarching PSE concepts (see Table 2 and Figure 2). Sixteen concepts were generated for specific pelvic pain conditions, including endometriosis and adenomyosis, bladder pain, vulvodynia/vulva pain, and sexual pain. Seven PSE concepts were generated for specific life stages, including “adolescent pelvic pain”, “pelvic pain during the reproductive years”, and “post-menopausal pelvic pain” (Table 3).

**Table 2:** The top three important PSE concepts for females with pelvic pain at the end of round 3, as grouped under overarching categories.

Overarching PSE categories (total number of concepts within each category)	Median importance rating^a^ (IQR)
The experience of pain (*n* = 5)	
All pain is real	6 (0)
Pain is personal	5 (1)
Pain is a normal bodily response	5 (1)
There are different types of pain (*n* = 2)	
There are differences between acute and persistent pain	5 (1)
There are differences between nociplastic and nociceptive pain (referring to IASP definitions)	5 (2.75)^b^
The brain and nervous system are involved in pain (*n* = 4)	
Pain is regulated/moderated by brain	6 (1)
Pain is an output of the brain	6 (1)
Pain is due to activity in the central nervous system	4 (1)
Pain is protective (*n* = 9)	
Feelings of safety can reduce pelvic pain	6 (1)
The brain places a high importance on the pelvis and, as a result, is highly protective	6 (2)^b^
The brain weighs up safety vs. danger	5 (1)
Persistent pelvic pain involves changes to the brain and nervous system (*n* = 7)	
Persistent pelvic pain involves a hypersensitive and overprotective pain system	6 (0)
The nervous system changes with persistent pain	6 (0.75)
Pain felt in one pelvic organ can lead to pain being felt elsewhere throughout the body (e.g., other pelvic organs, muscles)	6 (1)
Persistent pelvic pain and tissue pathology don't always correlate (*n* = 4)	
Pelvic pain is not an accurate marker of a worsening condition	6 (0)
Pain can occur in the absence of endometriosis lesions	6 (0)
Pelvic pain doesn't equate to tissue damage (i.e., pain can occur with and without pathology)	6 (0.75)
Persistent pelvic pain can change and improve (*n* = 3)	
Pelvic pain can change and improve	6 (0)
Pelvic pain is treatable	6 (0)
Our pain system is bioplastic	6 (1)
Many factors influence persistent pelvic pain (*n* = 3)	
Biopsychosocial factors influence pelvic pain and nervous system sensitivity	6 (1)
Pelvic pain is complex	6 (1)
Many factors influence pelvic pain. Pelvic pain can also influence these factors	6 (1)
Persistent pelvic pain can be influenced by biological factors (*n* = 24)	
Pelvic pain can be influenced by lifestyle factors (e.g., diet, exercise)	6 (1)
Pelvic pain can be influenced by sleep	6 (1)
Pain involves both peripheral and central contributors	6 (1)
Persistent pelvic pain can be influenced by the pelvic floor (*n* = 4)	
Increased tone in the pelvic floor can contribute to painful sex	6 (1)
Pelvic pain induces protective reflexes/guarding of the pelvic floor	6 (1)
Increased tone in the pelvic floor can occur without pelvic pain and pelvic pain can occur without increased tone in the pelvic floor	6 (1)
Persistent pelvic pain can be influenced by psychosocial factors (*n* = 19)	
Pelvic pain can be influenced by psychosocial factors (e.g., emotions, thoughts, and beliefs)	6 (0)
Unhelpful/negative thoughts can make pain worse	6 (1)
Pelvic pain can influence psychosocial symptoms	6 (1)
Persistent pelvic pain can be managed in many ways (*n* = 16)	
Active treatment strategies promote recovery	6 (0)
People can gain control over their pelvic pain	6 (0)
High self-efficacy can improve pelvic pain	6 (0.5)
Pain science education can help reduce persistent pelvic pain (*n* = 2)	
Pain science education can reduce anxiety, distress, and negative thoughts about pelvic pain	6 (0)
Pain science education can decrease nerve sensitisation	6 (1)

IASP, International Association for the Study of Pain; IQR, interquartile range; PSE, pain science education.

^a^ Median importance rating calculated from responses rated “not at all important” (1) – “very important” (6).

^b^ Consensus not reached.

**Table 3:** The top three important PSE concepts for specific pelvic pain conditions and life stages at the end of round 3.

Overarching PSE concept (total number of concepts within each category)	Median importance rating^a^ (IQR)
Endometriosis and adenomyosis (*n* = 8)	
Pelvic pain flares do not necessarily mean there are endometriosis lesions, nor recurrence	6 (0)
The amount of endometriosis seen laparoscopically does not correlate with the severity of symptoms, including pain	6 (0)
Adenomyosis can occur without pain	6 (0)
Bladder pain (*n* = 1)	
Peripheral and central contributors influence pelvic pain associated with bladder pain syndrome	5 (1.5)^b^
Vulvodynia/vulva pain (*n* = 4)	
Vulvodynia differs from vulvar pain secondary to pathology or disease	6 (1)
Vulvodynia is a complex primary pain condition without an identifiable nociceptive cause	5.5 (1)
Different inflammatory, genetic, hormonal, muscular factors may be involved in the development of vulvar pain	5.5 (1)
GPPPD (*n* = 3)	
Pelvic floor muscles can contribute to GPPPD	6 (0)
Understanding the anatomy, physiology, and normal function of the pelvis and pelvic organs can address misconceptions about GPPPD	6 (1)
Increased tone of the pelvic floor muscles is a protective reflex to avoid painful penetration or contact	6 (1)
Adolescent pelvic pain (*n* = 4)	
Dysmenorrhoea (period pain) that interferes with daily functioning is not normal	6 (0.75)
Early diagnosis and treatment are important for young people with period pain	6 (1)
Pain during adolescence may increase the risk for developing central sensitisation	6 (1)
Pelvic pain during the reproductive years (*n* = 1)	
Pelvic pain can be influenced by hormones and menstruation	6 (1)
Post-menopausal pelvic pain (*n* = 2)	
Pelvic pain can be influenced by hormones changing post-menopause	6 (1)
Pelvic pain can be influenced by physiological (e.g., structure and function of the pelvic region) and psychological effects of menopause	5.5 (1)

^a^ Median importance rating calculated from responses rated “not at all important” (1) – “very important” (6).

^b^ Consensus not reached.

Two (10%) panel members provided feedback on concept grouping and category names. One category name (“persistent pelvic pain doesn't always equate to tissue pathology”) was refined for clarity (“persistent pelvic pain and tissue pathology rarely correlate”). The full list of final concepts can be found in Supplementary File 2.

## 4 Discussion

This study gained consensus among an international panel of expert clinicians on important PSE concepts for females with pelvic pain. Using e-Delphi methodology, a panel of expert clinicians generated a final list of 125 concepts, of which 91 (72.8%) reached consensus for importance. These concepts may be integrated into educational resources and a curriculum to improve pelvic pain knowledge.

The PSE concepts developed in this study are similar to those valued by females with pelvic pain (16). For example, both consumers and clinicians have said that it is important for pelvic PSE to include content about changes to brain and nervous system with persistent pain, the influence of psychosocial contributors, and that pelvic pain can change and improve. Nevertheless, there are differences with how consumers and clinicians conceptualise and value the learning of biological contributors. Consumers report that it is important to recognise and validate pathological contributors of pelvic pain (e.g., endometriosis). Conversely, clinicians in this study de-emphasised the relationship between pathology and pelvic pain, rather they valued the learning that pain does not mean there is tissue damage. It is not surprising consumers value learning that pelvic pain can be an indication of pathology or disease given they are frequently dismissed and told that their pain is “all in their head” (25). The development of pelvic PSE should take into consideration consumers' perspectives, because without doing so, clinicians may provide education that is perceived as being dismissive or irrelevant (26, 27) – two key barriers in the uptake of pain education (7). Learning from the development of PSE for other pain conditions may provide valuable insights into how PSE for pelvic pain be implemented effectively into clinical practice.

The learning concepts generated in this study are similar to those that have been said to be important for other pain conditions. For example, learning concepts that emphasise all pain is real, that pain is not an accurate marker of tissue damage, pain is influenced by many factors, and persistent pain is overprotective have all been said to be important for people with complex regional pain syndrome (28) and musculoskeletal pain conditions (29–31), as well as adults (13, 14) and adolescents (32) with persistent pain. This study also identified important PSE concepts not identified for other pain conditions, including those emphasising the influence of culture, taboo, shame, and self-identity (see Supplementary File 2). Pelvic pain, and reproductive health broadly, are often stigmatised, contributing to patients and healthcare professionals being reluctant to discuss issues relating to the pelvis (33, 34), impeding people's health-seeking behaviours (35, 36) and ultimately their engagement with treatment strategies. Inclusion of these concepts in patient education may help destigmatise pelvic pain to improve clinical outcomes. Concepts identified as important for pelvic pain also included discussion about cross-organ sensitisation - a phenomenon observed in visceral persistent pain conditions, including pelvic pain (37–39). The exclusion of this concept from current PSE resources is not surprising because they have been primarily tailored to musculoskeletal pain conditions (6, 40), in which cross-organ sensitisation does not have a role. The inclusion of PSE concepts specific to pelvic pain conditions and life stages also differs compared to extant PSE curricula developed for other pain conditions. Whilst pelvic pain conditions do share similarities in pain mechanisms (e.g., peripheral and central sensitisation), there are also distinctions between different pelvic pain diagnoses. For example, endometriosis has a pathological contribution to pain whereas vulvodynia does not. The trajectory of pelvic pain also differs across age groups, including the prevalence of pelvic pain conditions (41, 42) and the factors that may have a role in pelvic pain (e.g., menstruation in those of reproductive age compared to hormonal changes associated with menopause) (42, 43). Delineating PSE concepts based on pelvic pain condition and life stage highlights the importance of tailoring education, not solely based on the umbrella term of pelvic pain itself, but on the individual person and their pain experience.

This study has strengths. This is the first-time consensus has been reached between expert clinicians on what should be included in PSE for females with pelvic pain. Clinical practice guidelines highlight patient education as a research priority (2, 3) and this study is an important first step in addressing this research gap. The PSE concepts generated in this study can also be used as a curriculum for healthcare professionals to implement in clinical practice. Further, concepts were included for specific pelvic pain conditions and life stages, which will assist with tailoring education to individuals; we recruited an international panel of expert clinicians across various healthcare professions, geographical locations, and expertise across healthcare sectors to ensure diverse responses and increase consensus validity; we determined consensus on learning concepts using robust criteria; as recommended in pain research a research protocol was lodged *a priori* (44).

This study has limitations. First, we did not aim to match concepts against empirical evidence. Some concepts included in the final list are not supported by current literature (e.g., “pain and dysfunction are often associated with imbalance”). The decision to include these concepts in the final results was because this study aimed to investigate what the clinicians value for inclusion in PSE, thus implying that they teach these concepts in their clinical practice. When developing an educational curriculum, it would be imperative that the content included would be based on empirical evidence or grounded in solid theories of pain. Second, defining the scope of the study to encompass only benign gynaecological and urological pelvic pain conditions resulted in no concepts being generated for gastrointestinal pelvic pain. Given gastrointestinal symptoms are common with pelvic pain, it would be imperative that PSE curricula include content on gastrointestinal contributions to pelvic pain. Third, the wording of these concepts was based upon use of language by the clinicians and would need to be adapted to be suitable for the educational level and health literacy of individual patients. Similarly, not all of these concepts would be applicable to every patient and would require clinician expertise to further tailor education to the individual. Last, the concepts generated were based on the primary researcher's interpretation of panel members' open-ended survey responses. Despite remaining close to the language used by the panel members, it is possible the researchers' interpretation of the concepts were inconsistent with the meaning intended by panel members; however, it is expected any misinterpretation of the concepts would have been clarified through the iterative survey rounds.

### 4.1 Research recommendations

Future research may consider co-creating PSE curricula with both clinicians and females with pelvic pain, in particular across the life stages identified (e.g., adolescence). Whilst this study proposes important PSE concepts, investigations are also needed into their wording and delivery to improve clinical applicability. Recent work emphasised the importance of enhancing clinicians' awareness of educational strategies for effectively implementing PSE concepts, along with providing simple resources and case scenario examples to support their application (45). Co-design research methods (46) with clinicians and consumers will be important for improving the clinical applicability of the concepts generated in this study. It would also be imperative to investigate the views of females with persistent pelvic pain to understand what educational content they value. The development of PSE resources for other pain conditions found that clinicians and consumers valued different learning concepts (14). Females with pelvic pain have highlighted the importance of acknowledging pathological contributions to pain (25, 47), whereas clinicians de-emphasised its importance in this study. These might be important to consider when developing PSE curricula for pelvic pain. There is also the need to develop a validated tool to assess pain knowledge specific to pelvic pain. Although tools have been developed to assess the knowledge of adults (48, 49) and children (50), none assess knowledge on concepts specific to pelvic pain. Finally, the efficacy of PSE could be tested in randomised clinical trials. Given that females with pelvic pain have expressed the importance of providing education alongside pain management interventions and the current evidence-base supports this, it would be beneficial to test its efficacy within a complex care package [e.g., alongside physiotherapy (51)], that could improve the outcomes of females with pelvic pain.

## 5 Conclusion

An interdisciplinary panel of expert clinicians identified 125 learning concepts important for female pelvic pain. For pelvic pain in general, 102 concepts were identified which were grouped under 13 overarching categories. Concepts were also identified for specific pelvic pain conditions and life stages. These concepts may inform the development of a PSE curriculum specifically tailored to females with pelvic pain. Future research may investigate the efficacy of such curriculum in empirical trials.

## Data Availability

The raw data supporting the conclusions of this article will be made available by the authors, without undue reservation.
