# Supplementary material for: Pain science education concepts for pelvic pain: an e-Delphi of expert clinicians
Source: Front Pain Res (Lausanne). 2025 Feb 4;6:1498996. doi: 10.3389/fpain.2025.1498996 (PMC11832528; doi:10.3389/fpain.2025.1498996)
Supplement: Supplementary file 1 [file Table1.docx]

**Supplementary File 1.** Content analysis inter-coder agreement

| **Cohen’s Kappa agreement threshold (Kappa value)** | **n (%) of codes** |
| --- | --- |
| No agreement (<0.20) | 7 (6.0) |
| Minimal agreement (0.21 and 0.39) | 0 (0.0) |
| Weak agreement (0.40 and 0.59) | 1 (0.1) |
| Moderate agreement (0.60 and 0.79) | 7 (6.0) |
| Strong agreement (0.80 and 0.90) | 3 (2.6) |
| Almost perfect (>0.90) | 98 (84.5) |
