# Supplementary material for: Pain science education concepts for pelvic pain: an e-Delphi of expert clinicians
Source: Front Pain Res (Lausanne). 2025 Feb 4;6:1498996. doi: 10.3389/fpain.2025.1498996 (PMC11832528; doi:10.3389/fpain.2025.1498996)
Supplement: Supplementary file 2 [file Table2.docx]

**Supplementary File 2.**

**Table 1.** Pain science education concepts for persistent pelvic pain

| **ROUND 2** | |  | **ROUND 3** | |  | **R2/3 stability** |  | **FINAL CONCEPTS** | |
| --- | --- | --- | --- | --- | --- | --- | --- | --- | --- |
| **Concept** | **Median importance rating* (IQR)** |  | **Concept** | **Median importance rating* (IQR)** |  | **p-value** |  | **Concept** | **Median importance rating* (IQR)** |
| **The experience of pain** | | | | | | | |  |  |
| All pain is real | 6 (0) |  | All pain is real | 6 (0) |  | 0.18 |  | All pain is real | 6 (0) |
| Pain is personal | 6 (0) |  | Pain is personal | 5 (1) |  | 0.615 |  | Pain is personal | 5 (1) |
| Pain is a normal bodily response | 6 (1) |  | Pain is a normal bodily response | 5 (1) |  | 0.856 |  | Pain is a normal bodily response | 5 (1) |
| Pain is an unpleasant sensory and emotional experience associated with, or resembling that associated with, actual or potential tissue damage | 5 (1) |  | Pain is an unpleasant sensory and emotional experience associated with, or resembling that associated with, actual or potential tissue damage | 5 (1) |  | 0.916 |  | Pain is an unpleasant sensory and emotional experience associated with, or resembling that associated with, actual or potential tissue damage | 5 (1) |
| Pain is an emergent bodily experience | 5 (2) |  | Pain is experienced in the body | 5 (2) |  | 0.777 |  | Pain is experienced in the body | 5 (2) |
| **There are different types of pain** | | | | | | | |  |  |
| There are differences between acute and persistent pain | 6 (1) |  | There are differences between acute and persistent pain | 6 (1) |  | 0.279 |  | There are differences between acute and persistent pain | 6 (1) |
| The differences between nociplastic and nociceptive pain | 5 (2) |  | The differences between nociplastic and nociceptive pain (referring to IASP definitions) | 5 (2.75) |  | 0.281 |  | There are differences between nociplastic and nociceptive pain (referring to IASP definitions) | 5 (2.75) |
| **The brain and nervous system are involved in pain** | | | | | | | |  |  |
| Pain is regulated/moderated by the brain | 6 (0) |  | Pain is regulated/moderated by the brain | 6 (1) |  | 0.942 |  | Pain is regulated/moderated by the brain | 6 (1) |
| Pain is an output of the brain | 6 (0) |  | Pain is an output of the brain | 6 (1) |  | 0.621 |  | Pain is an output of the brain | 6 (1) |
|  |  |  | Pain is due to activity in the central nervous system | 4 (1) |  | - |  | Pain is due to activity in the central nervous system | 4 (1) |
| The 'Neurotag' concept is one theoretical model of persistent pain | 4 (2) |  | The 'Neurotag' (ie. neural connections) concept is one theoretical model of persistent pain | 4 (2.25) |  | 0.813 |  | The 'Neurotag' (i.e. neural connections) concept is one theoretical model of persistent pain | 4 (2.25) |
| **Pain is protective** | | | | | | | |  |  |
| Feelings of safety can reduce pelvic pain | 6 (1) |  | Feelings of safety can reduce pelvic pain | 6 (1) |  | 1 |  | Feelings of safety can reduce pelvic pain | 6 (1) |
| Due to its importance in vital bodily functions and reproduction, the brain places a higher importance on the pelvis, and as a result, a higher level of protection | 5 (1) |  | Due to its importance in vital bodily functions and reproduction, the brain places a higher importance on the pelvis, and as a result, a higher level of protection | 6 (2) |  | 0.752 |  | The brain places a high importance on the pelvis and, as a result, is highly protective | 6 (2) |
| The brain weighs up safety vs danger | 6 (1) |  | The brain weighs up safety vs danger | 5 (1) |  | 0.167 |  | The brain weighs up safety vs danger | 5 (1) |
| There are danger sensors, not pain sensors | 6 (1) |  | There are danger sensors, not pain sensors | 5 (1) |  | 0.411 |  | There are danger sensors, not pain sensors | 5 (1) |
| Pain is a response to danger signals | 4.5 (2) |  | Pain is a response to danger signals | 5 (1.75) |  | 0.721 |  | Pain is a response to danger signals | 5 (1.75) |
| Pain is a prediction that the tissues are under threat | 5 (2.25) |  | Pain is a prediction that the tissues are under threat | 4.5 (2.5) |  | 0.782 |  | Pain is a prediction of potential threat to the tissues | 5 (2) |
| Pain is a predictor of potential threat | 5 (2.25) |  | Pain is a predictor of potential threat | 5 (2) |  | 0.371 |  |  |  |
|  |  |  | Persistent pain is our body doing too good of a job protecting us from potential threat | 5 (1.75) |  | - |  |  |  |
| Pain is a prediction that the tissues are not-fit-for-purpose | 4 (2) |  | Pain is a prediction that the tissues are not-fit-for-purpose | 4 (3.5) |  | 0.8 |  |  |  |
| Pain is predictive not responsive | 5 (2.25) |  | Pain is predictive not responsive | 5 (2) |  | 0.953 |  | Pain is predictive not responsive | 5 (2) |
| Pain is protective and promotes healing | 5 (1) |  | Pain is protective and promotes healing | 4.5 (1.75) |  | 0.831 |  | Pain is protective and promotes healing | 4.5 (1.75) |
| Pain flares are trying to tell you something | 4 (1) |  | Pain flares are trying to tell you something | 4 (1) |  | 0.601 |  | Pain flares are trying to tell you something | 4 (1) |
| **Persistent pelvic pain involves changes to the brain and nervous system** | | | | | | | |  |  |
| Persistent pelvic pain involves an overprotective pain system | 6 (1) |  | Persistent pelvic pain involves an overprotective pain system | 6 (0) |  | 0.943 |  | Persistent pelvic pain involves a hypersensitive and overprotective pain system | 6 (0) |
| When pain persists, the pain system becomes hypersensitive | 6 (0) |  | When pain persists, the pain system becomes hypersensitive | 6 (0.5) |  | 0.739 |  |  |  |
| The nervous system changes with persistent pain | 6 (0) |  | The nervous system changes with persistent pain | 6 (0) |  | 0.581 |  | The nervous system changes with persistent pain | 6 (0.75) |
| Pain involves nervous system maladaptation | 6 (1) |  | Persistent pain involves changes to the nervous system | 6 (1) |  | 0.952 |  |  |  |
| Pelvic pain can involve viscero-somatic pain referral | 6 (1) |  | Pelvic pain can involve viscero-somatic pain referral | 5 (1) |  | 0.327 |  | Pain felt in one pelvic organ can lead to pain being felt elsewhere in the body (e.g., other pelvic organs, muscles) | 6 (1) |
| Pelvic pain can involve viscero-visceral pain referral | 6 (0) |  | Pelvic pain can involve viscero-visceral pain referral | 6 (1) |  | 0.478 |  |  |  |
| Pelvic pain can involve pelvic organ crosstalk | 6 (0) |  | Pelvic pain can involve pelvic organ crosstalk | 5 (1) |  | 0.439 |  |  |  |
| Pain changes the brain | 6 (1) |  | Pain changes the brain | 6 (1) |  | 0.705 |  | Pain changes the brain | 6 (1) |
| Prolonged and repeated pain can prime or predict likelihood of pelvic pain | 6 (1) |  | Prolonged and repeated pain can prime or predict likelihood of pelvic pain | 6 (1) |  | 0.915 |  | Prolonged and repeated pelvic pain can prime or predict likelihood of persistent pelvic pain | 6 (1) |
| Peripheral and central sensitisation have a role in pain | 6 (1) |  | Peripheral and central sensitisation have a role in pain | 5 (1) |  | 0.476 |  | Peripheral and central sensitisation have a role in pain | 5 (1) |
| Pelvic pain can be widespread | 5 (2) |  | Pelvic pain can be widespread | 5.5 (1) |  | 0.26 |  | Pelvic pain may ‘spread’ | 5 (2) |
| Pelvic pain won't necessarily 'spread' | 4.5 (1.25) |  | Pelvic pain won't necessarily 'spread' | 5 (1) |  | 0.409 |  |  |  |
| **Persistent pelvic pain doesn't always equate to tissue pathology** | | | | | | | |  |  |
| Pelvic pain does not mean your condition is worsening | 6 (0) |  | Pelvic pain is not an accurate marker of a worsening condition | 6 (0) |  | 0.914 |  | Pelvic pain is not an accurate marker of a worsening condition | 6 (0) |
| Pain can occur in the absence of active endometriosis lesions | 6 (0) |  | Pain can occur in the absence of active endometriosis lesions | 6 (0) |  | 1 |  | Pain can occur in the absence of endometriosis lesions | 6 (0) |
| Pelvic pain can occur with or without pathology/tissue damage | 6 (0) |  | Pelvic pain can occur with or without pathology/tissue damage | 6 (0) |  | 0.705 |  | Pelvic pain doesn't equate to tissue damage (i.e., pain can occur with and without pathology) | 6 (0.75) |
| Pain doesn't equate to tissue damage | 6 (0) |  | Pain doesn't equate to tissue damage | 6 (1) |  | 0.603 |  |  |  |
| Pelvic pain is more than what can be ‘seen’ | 5 (2) |  | Pelvic pain is more than what can be ‘seen’ | 6 (1) |  | 0.672 |  | Pelvic pain is more than what can be ‘seen’ | 6 (1) |
| **Persistent pelvic pain can change and improve** | | | | | | | |  |  |
| Pelvic pain can change and improve | 6 (0) |  | Pelvic pain can change and improve | 6 (0) |  | 0.705 |  | Pelvic pain can change and improve | 6 (0) |
| Pelvic pain is treatable | 6 (0) |  | Pelvic pain is treatable | 6 (0) |  | 0.581 |  | Pelvic pain is treatable | 6 (0) |
| Our pain system is bioplastic | 6 (1) |  | Our pain system is bioplastic | 6 (1) |  | 0.546 |  | Our pain system is bioplastic | 6 (1) |
| **Many factors influence persistent pelvic pain** | | | | | | | |  |  |
| Biopsychosocial factors influence pain and nervous system sensitivity | 6 (0) |  | Biopsychosocial factors influence pain and nervous system sensitivity | 6 (0) |  | 0.655 |  | Biopsychosocial factors influence pelvic pain and nervous system sensitivity | 6 (1) |
| Pain depends on context | 6 (0) |  | Pain depends on context | 6 (1) |  | 0.461 |  |  |  |
| Pelvic pain is complex | 6 (1) |  | Pelvic pain is complex | 6 (1) |  | 0.477 |  | Pelvic pain is complex | 6 (1) |
| Many factors influence pelvic pain | 6 (1) |  | Many factors influence pelvic pain | 6 (1) |  | 0.317 |  | Many factors influence pelvic pain. Pelvic pain can also influence these factors. | 6 (1) |
| There is a bidirectional relationship of factors contributing to pelvic pain | 6 (1) |  | Pelvic pain is influenced by many factors. These factors can also be influenced by pelvic pain | 6 (1.5) |  | 0.78 |  |  |  |
| **Persistent pelvic pain can be influenced by biological factors** | | | | | | | |  |  |
| Pelvic pain can be influenced by lifestyle factors (e.g., diet, exercise) | 6 (1) |  | Pelvic pain can be influenced by lifestyle factors (e.g., diet, exercise) | 6 (0.5) |  | 0.458 |  | Pelvic pain can be influenced by lifestyle factors (e.g., diet, exercise) | 6 (1) |
| Pelvic pain can be influenced by exercise | 6 (1) |  | Pelvic pain can be influenced by exercise | 5 (1) |  | 0.564 |  |  |  |
| Pelvic pain can be influenced by diet | 6 (1) |  | Pelvic pain can be influenced by diet | 5 (1) |  | 0.43 |  |  |  |
| Pelvic pain can be influenced by sleep | 6 (0) |  | Pelvic pain can be influenced by sleep | 6 (1) |  | 0.589 |  | Pelvic pain can be influenced by sleep | 6 (1) |
| Pain involves both peripheral and central contributors | 6 (0) |  | Pain involves both peripheral and central contributors | 6 (1) |  | 0.058 |  | Pain involves both peripheral and central contributors | 6 (1) |
| There is an overlap between pelvic pain and other chronic conditions (e.g., fatigue) | 6 (0) |  | There is an overlap between pelvic pain and other chronic conditions (e.g., fatigue) | 6 (1) |  | 0.025 |  | There is an overlap between pelvic pain and other chronic conditions (e.g., fatigue) | 6 (1) |
| Ovulation can still occur with pelvic pain | 5 (2) |  | Ovulation can still occur with pelvic pain | 5.5 (2) |  | 0.799 |  | Ovulation can still occur with pelvic pain | 5.5 (2) |
|  |  |  | Menstruation/menstrual shedding is an inflammatory process and many of the substances involved in this process are nociceptor stimulants | 5 (0.75) |  | - |  | Menstruation/menstrual shedding is an inflammatory process and many of the substances involved in this process are nociceptor stimulants | 5 (0.75) |
| Dysmenorrhoea is not normal | 5.5 (1.25) |  | Dysmenorrhoea (period pain) is common but not normal | 5 (1) |  | 0.587 |  | Severe dysmenorrhoea (period pain) is common but not normal | 5 (1) |
| Pelvic pain can be influenced by the pelvic viscera | 6 (1) |  | Pelvic pain can be influenced by the pelvic viscera | 5 (1) |  | 0.774 |  | Pelvic pain can be influenced by the pelvic organs | 5 (1) |
| Pelvic pain can be influenced by menstruation | 6 (1) |  | Pelvic pain can be influenced by menstruation | 5 (1) |  | 0.48 |  | Pelvic pain can be influenced by menstruation | 5 (1) |
| Pelvic pain can be influenced by the musculoskeletal system | 6 (1) |  | Pelvic pain can be influenced by the musculoskeletal system | 5 (1) |  | 0.608 |  | Pelvic pain can be influenced by the musculoskeletal system | 5 (1) |
| Pelvic pain can be influenced by the hormonal system | 6 (1) |  | Pelvic pain can be influenced by the hormonal system | 5 (1) |  | 0.745 |  | Pelvic pain can be influenced by the hormonal system | 5 (1) |
| Pelvic pain does not prevent normal physiological function of the pelvis | 6 (1) |  | Pelvic pain does not prevent normal physiological function of the pelvis | 6 (1) |  | 0.722 |  | Pelvic pain may or may not prevent normal physiological function of the pelvis | 5 (1) |
| Pelvic pain can alter pelvic function | 5 (1) |  | Pelvic pain can alter pelvic function | 5.5 (1.75) |  | 0.758 |  |  |  |
| Understanding the anatomy, physiology, and normal function of the pelvis and pelvic organs is important | 6 (1) |  | Understanding the anatomy, physiology, and normal function of the pelvis and pelvic organs is important | 5 (1) |  | 0.272 |  | Understanding the anatomy, physiology, and normal function of the pelvis and pelvic organs is important to address pain misconceptions | 5 (1) |
| Pelvic pain can influence pain in other areas of the body | 6 (1) |  | Pelvic pain can influence pain in other areas of the body | 5 (1) |  | 0.19 |  | Pelvic pain can influence pain in other areas of the body | 5 (1) |
| The 'pill bottle' in your head can be used to improve pain | 6 (1) |  | The brain produces hormones that can help relieve pain | 5 (1) |  | 0.723 |  | The brain produces chemicals that can help relieve pain (e.g., endorphins) | 5 (1) |
| Pelvic pain is influenced by biological factors | 5 (1) |  | Pelvic pain is influenced by biological factors | 5 (1.5) |  | 0.572 |  | Pelvic pain is influenced by biological factors | 5 (1.5) |
| Nociception has a role in pain | 5 (1) |  | Nociception has a role in pain | 5 (1.5) |  | 0.732 |  | Nociception has a role in pelvic pain | 5 (1.5) |
| Pelvic pain can be influenced by inflammation | 5 (1) |  | Pelvic pain can be influenced by inflammation | 5 (1.75) |  | 0.81 |  | Pelvic pain can be influenced by inflammation | 5 (1.75) |
| Autonomic nervous system dysregulation influences not only pelvic function but also pain and inflammation | 5 (2) |  | Autonomic nervous system dysregulation influences not only pelvic function but also pain and inflammation | 5 (2) |  | 0.951 |  | Autonomic nervous system dysregulation influences not only pelvic function but also pain and inflammation | 5 (2) |
|  |  |  | Many pelvic pain conditions have no evidence of a nociceptive source | 5 (2) |  | - |  | Many pelvic pain conditions have no evidence of a nociceptive source | 5 (2) |
|  |  |  | Pelvic pain can alter pelvic function via increased sympathetic and decreased parasympathetic nervous system activity | 4.5 (1.75) |  | - |  | Pelvic pain can alter pelvic function via increased sympathetic and decreased parasympathetic nervous system activity | 4.5 (1.75) |
| Pelvic pain can be influenced by genetics | 4 (1) |  | Pelvic pain can be influenced by genetics | 4 (1) |  | 0.758 |  | Pelvic pain can be influenced by genetics and epigenetics | 4 (1) |
| Neurogenic inflammation has a role in pain | 5 (2) |  | Neurogenic inflammation has a role in pain | 4 (2) |  | 0.722 |  | Neurogenic inflammation has a role in pain | 4 (2) |
| Pain and dysfunction are often associated with imbalance | 4 (2) |  | Pain and dysfunction are often associated with imbalance | 4 (2) |  | 0.408 |  | Pain and dysfunction are often associated with imbalance | 4 (2) |
| **Persistent pelvic pain can be influenced by the pelvic floor** | | | | | | | |  |  |
| An overactive pelvic floor can contribute to painful sex | 6 (1) |  | An overactive pelvic floor can contribute to painful sex | 6 (1) |  | 0.832 |  | Increased tone in the pelvic floor can contribute to painful sex | 6 (1) |
| Pelvic pain induces protective reflexes/guarding of the pelvic floor | 5 (1) |  | Pelvic pain induces protective reflexes/guarding of the pelvic floor | 6 (1) |  | 0.952 |  | Pelvic pain induces protective reflexes/guarding of the pelvic floor | 6 (1) |
|  |  |  | An overactive pelvic floor can occur without pain and pain can occur without an overactive pelvic floor | 6 (1) |  | - |  | Increased tone in the pelvic floor can occur without pelvic pain and pelvic pain can occur without increased tone in the pelvic floor | 6 (1) |
| Overactive pelvic floor is a symptom of pelvic pain not a cause | 5 (3) |  | Overactive pelvic floor is a symptom of pelvic pain not a cause | 4.5 (1.25) |  | 0.269 |  | Increased tone in the pelvic floor muscles may contribute to pelvic pain and vice versa, with multisystem drivers involved | 5 (2) |
|  |  |  | Overactive pelvic floor muscles can contribute to pelvic pain and vice versa, with multisystemic drivers involved | 5 (1.75) |  | - |  |  |  |
| **Persistent pelvic pain can be influenced by psychosocial factors** | | | | | | | |  |  |
| Pelvic pain can be influenced by psychosocial factors (e.g., thoughts, feelings) | 6 (0) |  | Pelvic pain can be influenced by psychosocial factors (e.g., thoughts, feelings) | 6 (0) |  | 1 |  | Pelvic pain can be influenced by psychosocial factors (e.g., emotions, thoughts, and beliefs) | 6 (0) |
| Pelvic pain can be influenced by emotions, thoughts, and beliefs | 6 (0) |  | Pelvic pain can be influenced by emotions, thoughts, and beliefs | 6 (0.5) |  | 0.48 |  |  |  |
| Catastrophising can make pain worse | 6 (0) |  | Unhelpful/negative thoughts can make pain worse | 6 (1) |  | 0.792 |  | Unhelpful/negative thoughts can make pain worse | 6 (1) |
| Pelvic pain can be influenced by mood | 6 (1) |  | Pelvic pain can be influenced by mood | 6 (1) |  | 0.305 |  | Pelvic pain can be influenced by mood | 6 (1) |
| Pelvic pain can be influenced by stress | 6 (1) |  | Pelvic pain can be influenced by stress | 6 (1) |  | 0.417 |  | Pelvic pain can be influenced by stress | 6 (1) |
| Pelvic pain can be influenced by depression and anxiety | 6 (1) |  | Pelvic pain can be influenced by depression and anxiety | 6 (1) |  | 0.755 |  | Pelvic pain can be influenced by depression and anxiety | 6 (1) |
| Pelvic pain can be influenced by trauma | 6 (1) |  | Pelvic pain can be influenced by trauma | 6 (1) |  | 0.751 |  | Pelvic pain can be influenced by trauma | 6 (1) |
| Pelvic pain can influence psychosocial symptoms | 6 (1) |  | Pelvic pain can influence psychosocial symptoms | 6 (1) |  | 0.539 |  | Pelvic pain can influence psychosocial symptoms | 6 (1) |
| Pelvic pain affects sexual function, libido, self-touch, and relationships | 6 (1) |  | Pelvic pain affects sexual function, libido, self-touch, and relationships | 6 (1) |  | 0.942 |  | Pelvic pain can affect sexual function, libido, self-touch, and relationships | 6 (1) |
| Fear-avoidance can make pain worse | 6 (1) |  | Fear-avoidance can make pain worse | 6 (1.5) |  | 0.25 |  | Fear-avoidance can make pain worse | 6 (1.5) |
|  |  |  | Pelvic pain can be influenced by previous sexual abuse | 5.5 (1) |  | - |  | Pelvic pain can be influenced by previous sexual abuse | 5.5 (1) |
| Pelvic pain can be influenced by identity | 5 (2) |  | Pelvic pain can be influenced by how one views one’s self | 5 (0.75) |  | 0.711 |  | Pelvic pain can be influenced by how one views one’s self | 5 (0.75) |
| Pelvic pain can be influenced by shame | 5.5 (1.25) |  | Pelvic pain can be influenced by shame | 5 (1) |  | 0.574 |  | Pelvic pain can be influenced by shame | 5 (1) |
| Adverse early life experiences increase vulnerability to pelvic pain | 6 (1) |  | Adverse early life experiences increase vulnerability to pelvic pain | 5 (1) |  | 0.951 |  | Adverse early life experiences increase vulnerability to pelvic pain | 5 (1) |
| A person's history can influence their pelvic pain | 6 (1) |  | A person's history can influence their pelvic pain | 5 (1.5) |  | 0.49 |  | A person's history can influence their pelvic pain | 5 (1.5) |
| Pelvic pain can be influenced by stigma | 5.5 (2) |  | Pelvic pain can be influenced by stigma | 5 (1.5) |  | 0.876 |  | Pelvic pain can be influenced by stigma | 5 (1.5) |
| Knowledge of the pelvis reduces emotional burden and taboo associated with pelvic pain | 5 (1) |  | Knowledge of the pelvis reduces emotional burden and taboo associated with pelvic pain | 5 (2) |  | 0.264 |  | Knowledge of the pelvis reduces emotional burden and taboo associated with pelvic pain | 5 (2) |
| Pelvic pain can be influenced by injustice | 5 (2) |  | Pelvic pain can be influenced by injustice | 5 (2) |  | 0.832 |  | Pelvic pain can be influenced by injustice | 5 (2) |
| Pelvic pain has educational, cultural, and social implications | 5 (1) |  | Pelvic pain has educational, cultural, and social implications | 5 (2) |  | 0.377 |  | Pelvic pain can have educational, cultural, and social implications | 5 (2) |
| Pelvic pain can be influenced by isolation | 5 (1) |  | Pelvic pain can be influenced by isolation | 4.5 (1.75) |  | 0.491 |  | Pelvic pain can be influenced by isolation | 4.5 (1.75) |
| **Persistent pelvic pain can be managed in many ways** | | | | | | | |  |  |
| Active treatment strategies promote recovery | 6 (0) |  | Active treatment strategies promote recovery | 6 (0) |  | 0.491 |  | Active treatment strategies promote recovery | 6 (0) |
| You have control over your pain | 6 (1) |  | People can gain control over their pelvic pain | 6 (0) |  | 0.891 |  | People can gain control over their pelvic pain | 6 (0) |
| High self-efficacy can improve pelvic pain | 6 (0) |  | High self-efficacy can improve pelvic pain | 6 (0.5) |  | 1 |  | High self-efficacy can improve pelvic pain | 6 (0.5) |
| There are a wide range of treatments for pelvic pain | 6 (1) |  | There are a wide range of treatments for pelvic pain | 6 (1) |  | 0.794 |  | There are a wide range of treatments for pelvic pain | 6 (1) |
| Look at the big picture when treating pelvic pain | 6 (1) |  | Look at the big picture when treating pelvic pain | 6 (1) |  | 0.587 |  | Removed Round 3 (irrelevant) |  |
| Early treatment is important to reduce sensitisation | 6 (1) |  | Early treatment is important to reduce sensitisation | 6 (1) |  | 0.805 |  | Early treatment is important to reduce sensitisation | 6 (1) |
| Pacing can help improve pain | 6 (1) |  | Pacing can help improve pain | 6 (1) |  | 0.19 |  | Pacing can help improve pain | 6 (1) |
| Changing behaviours and beliefs can improve pelvic pain | 6 (1) |  | Changing behaviours and beliefs can improve pelvic pain | 6 (1) |  | 0.627 |  | Changing behaviours and beliefs can improve pelvic pain | 6 (1) |
| Exercise can improve pelvic pain | 6 (0) |  | Exercise can improve pelvic pain | 6 (1) |  | 0.317 |  | Exercise can improve pelvic pain | 6 (1) |
| It is important to change boom-bust behaviours | 6 (0) |  | It is important to change boom-bust behaviours (i.e., Doing too much one day resulting in increased pain the following day) | 6 (1) |  | 0.279 |  | It is important to change boom-bust behaviours (i.e., doing too much one day resulting in increased pain the following day) | 6 (1) |
| Pelvic pain should be managed holistically | 6 (1) |  | Pelvic pain should be managed holistically | 5.5 (1) |  | 0.337 |  | Pelvic pain should be managed holistically | 5.5 (1) |
|  |  |  | The purposeful pursuit of pleasure is an important part of self-efficacy and recovery | 5.5 (1) |  | - |  | The purposeful pursuit of pleasure is an important part of self-efficacy and recovery | 5.5 (1) |
| Interventions can affect pain modulation | 5 (0.25) |  | Interventions can affect pain modulation | 5.5 (1) |  | 0.228 |  | Interventions can affect pain modulation | 5.5 (1) |
| Referred pain can be managed as one pain condition | 5 (1) |  | Pain felt in other parts of the body can be managed as one pain condition | 5.5 (2) |  | 0.537 |  | Pain felt in other parts of the body can be managed as one pain condition | 5.5 (2) |
| Pelvic pain should be managed using multi-modal interventions | 6 (0) |  | Pelvic pain should be managed using multi-modal interventions | 5 (1) |  | 0.627 |  | Pelvic pain should be managed using multi-modal interventions | 5 (1) |
| Painful techniques used as treatment can increase pain | 6 (1) |  | Painful techniques used as treatment can increase pain | 5 (1.75) |  | 0.23 |  | Painful techniques used as treatment can increase pain | 5 (1.75) |
| Better connections between the brain and the pelvis (both motor and sensory) can help overcome pain | 5 (2) |  | Better connections between the brain and the pelvis (both motor and sensory) can help overcome pain | 5 (2) |  | 0.717 |  | Better connections between the brain and the pelvis (both motor and sensory) can help overcome pain | 5 (2) |
| Certain activities have implications on an overactive core | 5 (1) |  | Certain activities have implications on an overactive core | 4 (2) |  | 0.414 |  | Removed Round 3 (irrelevant) |  |
| **Pain science education can help reduce persistent pelvic pain** | | | | | | | |  |  |
| Pain science education can reduce anxiety, distress, and negative thoughts about pelvic pain | 6 (0) |  | Pain science education can reduce anxiety, distress, and negative thoughts about pelvic pain | 6 (0) |  | 0.671 |  | Pain science education can reduce anxiety, distress, and negative thoughts about pelvic pain | 6 (0) |
| Pain science education can decrease nerve sensitisation | 6 (1) |  | Pain science education can decrease nerve sensitisation | 6 (1) |  | 1 |  | Pain science education can decrease nerve sensitisation | 6 (1) |

Shading = IQR not meeting pre-defined consensus criteria

Shading = Stability of response distribution not reached between survey rounds

Shading = re-worded by panel members

Shading = re-worded by research team for clarity

Shading = concepts removed

| **ROUND 2** | |  | **ROUND 3** | |  | **R2/3 stability** |  | **FINAL CONCEPTS** | |
| --- | --- | --- | --- | --- | --- | --- | --- | --- | --- |
| **Concept** | **Median importance rating* (IQR)** |  | **Concept** | **Median importance rating* (IQR)** |  | **p-value** |  | **Concept** | **Median importance rating* (IQR)** |
| **Endometriosis/adenomyosis** | | | | | | | | | |
| Pelvic pain flares do not necessarily mean there are active endometriosis lesions, nor recurrence | 6 (0) |  | Pelvic pain flares do not necessarily mean there are active endometriosis lesions, nor recurrence | 6 (0) |  | 1 |  | Pelvic pain flares do not necessarily mean there are endometriosis lesions, nor recurrence | 6 (0) |
|  |  |  | The amount of endometriosis seen laparoscopically does not correlate with the severity of symptoms, including pain | 6 (0) |  | - |  | The amount of endometriosis seen laparoscopically does not correlate with the severity of symptoms, including pain | 6 (0) |
|  |  |  | Adenomyosis can occur without pain | 6 (0) |  | - |  | Adenomyosis can occur without pain | 6 (0) |
| Endometriosis-associated pelvic pain is multidimensional | 6 (0.25) |  | Endometriosis-associated pelvic pain is multidimensional | 6 (1) |  | 0.161 |  | Endometriosis-associated pelvic pain is multidimensional | 6 (1) |
| Early experiences of prolonged or repeated pain, such as in endometriosis, can prime the pain system and are predictors for experiencing persistent pelvic pain | 6 (1) |  | Early experiences of prolonged or repeated pain, such as in endometriosis, can prime the pain system and are predictors for experiencing persistent pelvic pain | 6 (1) |  | 0.366 |  | Early experiences of prolonged or repeated pain, such as in endometriosis, can prime the pain system and are predictors for experiencing persistent pelvic pain | 6 (1) |
| Endometriosis can involve nociplastic and neuropathic pain | 5.5 (1) |  | Endometriosis can involve nociplastic and neuropathic pain | 5.5 (2.75) |  | 0.224 |  | Endometriosis can involve nociplastic and neuropathic pain | 5.5 (2.75) |
| Peripheral contributors influence adenomyosis-associated pelvic pain | 5 (1) |  | Peripheral contributors influence adenomyosis-associated pelvic pain | 5 (1.75) |  | 0.237 |  | Adenomyosis-associated pain can be influenced by stimuli in the periphery of the body | 5 (1.75) |
| Peripheral contributors influence endometriosis-associated pelvic pain | 5 (1) |  | Peripheral contributors influence endometriosis-associated pelvic pain | 5.5 (1.75) |  | 0.661 |  | Endometriosis-associated pain can be influenced by stimuli in the periphery of the body | 5 (2) |
| Endometriosis can involve peripheral nociception | 5 (1) |  | Endometriosis can involve peripheral nociception | 4.5 (2) |  | 0.326 |  |  |  |
| **Bladder pain** | | | | | | | | |  |
| Peripheral contributors influence pelvic pain associated with interstitial cystitis/bladder pain syndrome | 5 (1) |  | Peripheral contributors influence pelvic pain associated with bladder pain syndrome | 5 (1.5) |  | 0.638 |  | Peripheral and central contributors influence pelvic pain associated with bladder pain syndrome | 5 (1.5) |
| Hypnotherapy can target nerve sensitisation | 4 (2) |  | Removed Round 2 (irrelevant) |  |  |  |  |  |  |
| **Vulvodynia/vulva pain** | | | | | | | | |  |
|  |  |  | Vulvodynia can coexist with other chronic conditions (e.g., thrush, lichen sclerosis) | 6 (1) |  | - |  | Vulvodynia can coexist with other chronic conditions (e.g., thrush, lichen sclerosis) | 6 (1) |
| Vulvodynia is a complex primary pain condition without an identifiable nociceptive cause | 6 (1) |  | Vulvodynia is a complex primary pain condition without an identifiable nociceptive cause | 5.5 (1) |  | 0.809 |  | Vulvodynia is a complex primary pain condition without an identifiable nociceptive cause | 5.5 (1) |
| Different inflammatory, genetic, hormonal, muscular factors may be involved in the development of vulvar pain | 5 (1) |  | Different inflammatory, genetic, hormonal, muscular factors may be involved in the development of vulvar pain | 5.5 (1) |  | 0.582 |  | Different inflammatory, genetic, hormonal, muscular factors may be involved in the development of vulvar pain | 5.5 (1) |
| Vulvodynia differs from vulvar pain secondary to pathology or disease | 5 (1.25) |  | Vulvodynia differs from vulvar pain secondary to pathology or disease | 5 (1) |  | 0.958 |  | Vulvodynia differs from vulvar pain secondary to pathology or disease | 5 (1) |
| Hypnotherapy can target nerve sensitisation | 4 (1.75) |  | Removed Round 2 (irrelevant) |  |  |  |  |  |  |
| **Genito-pelvic pain/penetration disorder (GPPPD)** | | | | | | | | |  |
| Pelvic floor muscles can contribute to GPPPD | 6 (1) |  | Pelvic floor muscles can contribute to GPPPD | 6 (0) |  | 0.483 |  | Pelvic floor muscles can contribute to GPPPD | 6 (0) |
|  |  |  | Understanding the anatomy, physiology, and normal function of the pelvis and pelvic organs can address misconceptions about GPPPD | 6 (1) |  | - |  | Understanding the anatomy, physiology, and normal function of the pelvis and pelvic organs can address misconceptions about GPPPD | 6 (1) |
|  |  |  | Overactivity of the pelvic floor muscles is a protective reflex to avoid painful penetration or contact | 6 (1) |  | - |  | Increased tone of the pelvic floor muscles is a protective reflex to avoid painful penetration or contact | 6 (1) |

**Table 2.** Pain science education concepts for persistent pelvic pain diagnoses

Shading = IQR not meeting pre-defined consensus criteria

Shading = re-worded by panel members

Shading = re-worded by research team for clarity

Shading = concepts removed

| **ROUND 2** | |  | **ROUND 3** | |  | **R2/3 stability** |  | **FINAL CONCEPTS** | |
| --- | --- | --- | --- | --- | --- | --- | --- | --- | --- |
| **Concept** | **Median importance rating* (IQR)** |  | **Concept** | **Median importance rating* (IQR)** |  | **p-value** |  | **Concept** | **Median importance rating* (IQR)** |
| **Adolescent pelvic pain** | | | | | | | | | |
| Dysmenorrhoea is not normal | 6 (0.25) |  | Dysmenorrhoea (period pain) that interferes with daily functioning is not normal | 6 (0.75) |  | 0.518 |  | Dysmenorrhoea (period pain) that interferes with daily functioning is not normal | 6 (0.75) |
| Young people with menstrual pain should be diagnosed and treated early | 6 (0) |  | Young people with menstrual pain should be diagnosed and treated early | 6 (1) |  | 0.679 |  | Early diagnosis and treatment are important for young people with period pain | 6 (1) |
| Some pelvic pain conditions that affect adults (e.g., endometriosis and adenomyosis) can be reversed in adolescence | 5.5 (1) |  | Some pelvic pain conditions that affect adults (e.g., endometriosis and adenomyosis) can be reversed in adolescence | 6 (1) |  | 0.964 |  | Pelvic pain experienced in adolescence can be treated effectively | 6 (1) |
| Pain during adolescence may increase the risk for developing centralized pain | 5 (1) |  | Pain during adolescence may increase the risk for developing centralized pain | 6 (1) |  | 1 |  | Pain during adolescence may increase the risk for developing central sensitisation | 6 (1) |
| **Pelvic pain during the reproductive years** | | | | | | | | |  |
| Pelvic pain can be influenced by hormones and menstruation | 6 (1) |  | Pelvic pain can be influenced by hormones and menstruation | 6 (1) |  | 1 |  | Pelvic pain can be influenced by hormones and menstruation | 6 (1) |
| **Post-menopausal pelvic pain** | | | | | | | | |  |
| Pelvic pain can be influenced by physiological (e.g., structure and function of the pelvic region) and psychological effects of menopause | 5 (1) |  | Pelvic pain can be influenced by physiological (e.g., structure and function of the pelvic region) and psychological effects of menopause | 6 (1) |  | 0.305 |  | Pelvic pain can be influenced by physiological (e.g., structure and function of the pelvic region) and psychological effects of menopause | 6 (1) |
| Pelvic pain can be influenced by the lack of hormones post-menopause | 5 (1) |  | Pelvic pain can be influenced by hormones changing post-menopause | 5.5 (1) |  | 0.852 |  | Pelvic pain can be influenced by hormones changing post-menopause | 5.5 (1) |

**Table 3.** Pain science education concepts for persistent pelvic pain at different life stages

Shading = re-worded by panel members

Shading = re-worded by research team for clarity
